# Impact of taxane-based chemotherapeutics on male reproductive function

**DOI:** 10.1530/RAF-22-0134

**Published:** 2023-03-28

**Authors:** Charvi Kanodia, Michael P Rimmer, Kathleen Duffin, Rod T Mitchell

**Affiliations:** 1 Edinburgh Medical School, University of Edinburgh, UK; 2 MRC Centre for Reproductive Health, Institute of Regeneration and Repair, University of Edinburgh, UK; 3 Biomedical Sciences, University of Edinburgh, UK

**Keywords:** fertility preservation, fertility, taxanes, chemotherapy, paclitaxel

## Abstract

Men and boys with cancer treated with chemotherapy are known to have reduced fertility following their treatment. This is because some chemotherapy drugs can damage the cells in the testicles that make sperm. This study found there is limited information available on the effect of one group of chemotherapy drugs, called taxanes, on testicular function and fertility. More studies are needed to aid clinicians in advising patients on how this taxane-based chemotherapy may affect their future fertility.

Chemotherapy exposure may reduce fertility in males. Adult men may cryopreserve sperm prior to commencing cancer therapy; however, for pre-pubertal males who do not produce sperm, fertility preservation remains experimental. At present, no human has ever been born from cryopreserved pre-pubertal testis tissue, with the most recent breakthrough in this area being the birth of a Rhesus Macaque following autologous transplant of cryopreserved pre-pubertal testis tissue (Fayomi *et al.* 2019). Current clinical practice on male pre-pubertal fertility preservation varies globally in terms of eligibility for tissue cryopreservation, methods and duration of storage, future clinical use, and consensus on the assessment of tissue function and reproductive outcomes.

Although numerous chemotherapeutics are used to treat cancers, the impact of different agents on reproductive function and fertility is poorly understood. Taxane-based chemotherapeutics are used to treat numerous cancers; however, robust data on their impact on male fertility is lacking. We reviewed the literature on the effects of taxane-based chemotherapy in male patients on subsequent gonadal function and fertility.

We systematically searched PubMed and Scopus using a previously published methodology (Tian *et al.* 2020) and registered our protocol (PROSPERO-CRD42021296306). The search terms used in this review are outlined in Supplementary Table 1 (see section on supplementary materials given at the end of this article). We included studies reporting the effects of taxane-based chemotherapy on testicular development and function using PRISMA guidelines. We identified 458 studies, of which 87 were assessed for eligibility (Supplementary Table 2). Five studies met inclusion criteria (Fig. 1) and involved 512 patients, with 94 eligible for this review (male patients receiving taxanes), and the characteristics of these studies can be found in Table 1.

**Figure 1:**
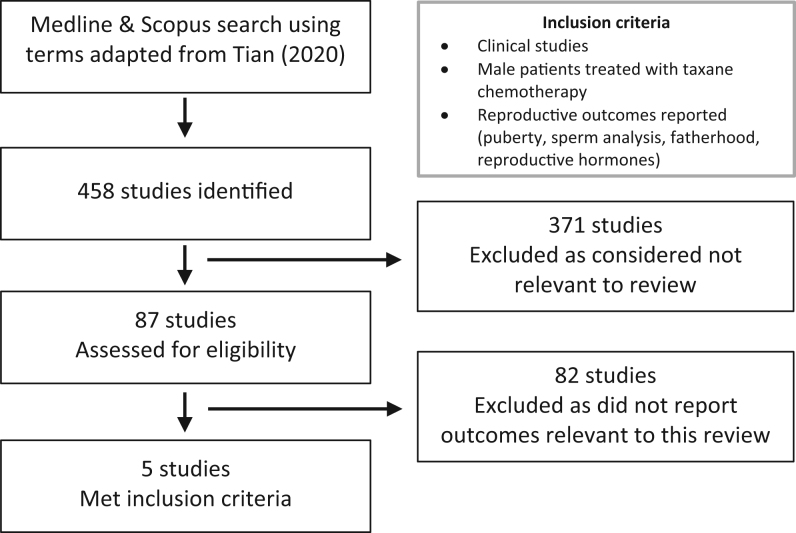
PRISMA flow diagram.

**Table 1:** Studies which met inclusion criteria. Prepubertal patients were not included.

Reference	Study design	Study aim	Outcome measures*	Patients, *n*		Summary of results	Commentary
				Total	Eligible**		
Pectasides *et al.* 2009	Controlled clinical study	To investigate the effect of methotrexate, paclitaxel, ifosfamide, and cisplatin on fertility in poor-risk nonseminomatous germ cell tumours	Testosterone, FSH, LH, sperm analysis, fatherhood	30	30	The majority of men demonstrated recovery of spermatogenesis. Leydig cell function was not affected.	All patients received paclitaxel
Chatzidarellis *et al.* 2010	Controlled clinical study	To assess the effects of taxanes on the male reproductive axis	FSH, LH, inhibin B, testicular volume	40	40	Following taxane chemotherapy, there was a significant reduction in inhibin B, increase in FSH, and decrease in bilateral testicular volume, with no significant change in LH.	All patients received paclitaxel
Strasser *et al.* 2006	Cross-sectional study	To assess whether hypogonadism and autonomic dysfunction contribute to fatigue, decreased sexual desire, and depression in patients with incurable cancer	Testosterone, FSH, LH	48	23	64% had hypogonadism and this was associated with fatigue and negative mood.	23 patients had paclitaxel; results were not stratified by treatment
Burney *et al.* 2012	Cross-sectional study	To determine the relationship between testosterone levels, inflammation, and symptom burden in male cancer patients	Testosterone	95	12	Testosterone levels were lower in patients with cancer cachexia than non-cachexia and non-cancer controls	12 patients received taxane chemotherapy; results were not stratified by treatment.
Rothermundt *et al.* 2018	Cohort study	To present baseline characteristics and treatment strategies for patients with primary or relapsed germ cell tumours	Testosterone	299	1	7.3% of seminoma and 5.6% of non-seminoma patients had low testosterone levels	One patient received paclitaxel; results were not stratified by treatment.

*Outcome measures are only listed if relevant to this review. **Patients were eligible if they were male, treated with taxane chemotherapy, and had one or more reproductive outcome reported.

All 5 studies reported testosterone levels, 3/5 reported gonadotrophin levels, and 1/5 reported fatherhood, sperm count/analysis, inhibin B, and testicular volume.

Two studies (Pectasides *et al.* 2009, Chatzidarellis *et al.* 2010) specifically reported reproductive outcomes in patients who received taxane chemotherapy with all patients (*n* = 70) in these studies receiving paclitaxel and additional chemotherapy, and neither study included a control group.

Chatzidarellis *et al.* reported 40 male patients (aged 28 and 60 years) receiving paclitaxel or docetaxel; identifying a significant reduction in inhibin B, an increase in follicle-stimulating hormone (FSH), and a decrease in bilateral testicular volume, with no significant change in luteinizing hormone (LH) (Chatzidarellis *et al.* 2010). Whilst information about the total taxane dose per patient is provided, outcome measures are not reported for individual patients.

Pectasides *et al.* report on 30 male patients (aged 17–62 years) treated with paclitaxel, methotrexate, ifosfamide, and cisplatin for poor-risk non-seminoma germ cell tumours; outcome measures were available for 21 patients. Serum FSH levels were significantly elevated 12 months after treatment completion; however, they returned to normal levels at 18 months post-treatment. LH and testosterone levels were unaffected. Overall, 17/21 (80.9%) patients demonstrated recovery of spermatogenesis post-treatment, and a total of 5 patients had gone on to father children.

For the remaining three studies, 24/442 patients received taxane chemotherapy. However, no studies report outcomes of individual patients or stratified by treatment received (Strasser *et al.* 2006, Burney *et al.* 2012, Rothermundt *et al.* 2018).

Limited data are available on taxane-based chemotherapy and reproductive outcomes, with no data available for prepubertal males. The single study reporting on spermatogenesis showed that sperm production resumed in the majority of patients (Pectasides *et al.* 2009), while studies reporting endocrine function post-taxane treatment demonstrated elevated FSH (Pectasides *et al.* 2009, Chatzidarellis *et al.* 2010) and reduced inhibin-B (Chatzidarellis *et al.* 2010). The impact of specific dosing regimens or duration of treatment on reproductive outcomes could not be determined. Prospective data collection on endocrine function, semen analysis, and fatherhood for males receiving taxane-based chemotherapy is required in order to inform clinicians when counselling patients receiving these chemotherapeutics on their future fertility. Given the experimental nature of fertility preservation for pre-pubertal males, the importance of understanding how taxane-based chemotherapy can impact future fertility cannot be understated.

## Supplementary Material

Supplementary Table 1 – Search terms and strategy for identification of publications relating to fertility outcomes after taxane-based chemotherapy in childhood cancer survivors. Search strategy adapted from Tian 2020.

Supplementary Table 2: Full text articles screened and excluded

## Declaration of interest

The authors declare that there is no conflict of interest that could be perceived as prejudicing the impartiality of this review.

## Funding

MPR and RTM are funded by an MRC Centre for Reproductive Health Grant No: MR/N022556/1. RTM is funded by a UK Research and Innovation (UKRI) Future Leaders Fellowship MR/S017151/1. KD is funded by a CRUK grant (C157/A25193).

## Author contribution statement

CK, MPR and RTM conceived the idea for the article. MPR and KD undertook the literature search. MPR, CK and KD screened abstracts, extracted data and wrote the manuscript. MPR produced tables and figures. CK, MPR, KD and RTM wrote the article and approved it for submission.

## References

[bib1] Burney BO Hayes TG Smiechowska J Cardwell G Papusha V Bhargava P Konda B Auchus RJ & Garcia JM 2012 Low testosterone levels and increased inflammatory markers in patients with cancer and relationship with cachexia. Journal of Clinical Endocrinology and Metabolism 97 E700–E709. (10.1210/jc.2011-2387) 22419719

[bib2] Chatzidarellis E Makrilia N Giza L Georgiadis E Alamara C & Syrigos KN 2010 Effects of taxane-based chemotherapy on inhibin B and gonadotropins as biomarkers of spermatogenesis. Fertility and Sterility 94 558–563. (10.1016/j.fertnstert.2009.03.068) 19765702

[bib3] Fayomi AP Peters K Sukhwani M Valli-Pulaski H Shetty G Meistrich ML Houser L Robertson N Roberts V Ramsey C, 2019 Autologous grafting of cryopreserved prepubertal rhesus testis produces sperm and offspring. Science 363 1314–1319. (10.1126/science.aav2914) 30898927 PMC6598202

[bib4] Pectasides D Pectasides E Papaxoinis G Skondra M Gerostathou M Karageorgopoulou S Kamposioras C Tountas N Koumarianou A Psyrri A, 2009 Testicular function in poor-risk nonseminomatous germ cell tumors treated with methotrexate, paclitaxel, ifosfamide, and cisplatin combination chemotherapy. Journal of Andrology 30 280–286. (10.2164/jandrol.108.006437) 19136393

[bib5] Rothermundt C Thurneysen C Cathomas R Müller B Mingrone W Hirschi-Blickenstorfer A Wehrhahn T Ruf C Rothschild S Seifert B, 2018 Baseline characteristics and patterns of care in testicular cancer patients: first data from the Swiss Austrian German Testicular Cancer Cohort Study (SAG TCCS). Swiss Medical Weekly 148 w14640. (10.4414/smw.2018.14640) 30044478

[bib6] Strasser F Palmer JL Degracia B Willey JS Schover LR Yusuf SW Pisters K Vassilopoulou-Sellin R & Bruera E 2006 The impact of hypogonadism and autonomic dysfunction on fatigue, emotional function, and sexual desire in male with advanced center: a pilot study. Cancer 107 2949–2957. (10.1200/JCO.2005.05.1847) 17103445

[bib7] Tian EN Brougham M Wallace WHB & Mitchell RT 2020. Impacts of platinum-based chemotherapy on subsequent testicular function and fertility in boys with cancer. Human Reproduction Update 26 874–885. (10.1093/humupd/dmaa041) 32935838 PMC7600277

