# Supplementary material for: Impact of taxane-based chemotherapeutics on male reproductive function
Source: Reprod Fertil. 2023 Mar 28;4(1):e220134. doi: 10.1530/RAF-22-0134 (PMC10083651; doi:10.1530/RAF-22-0134)
Supplement: Supplementary Table 1 – Search terms and strategy for identification of publications relating to fertility outcomes after taxane-based chemotherapy in childhood cancer survivors. Search strategy adapted from Tian 2020. [file supplementary_table_1.pdf]

Supplementary Table 1 – Search terms and strategy for identification of publications relating to fertility outcomes after taxane-based chemotherapy in childhood cancer survivors. Search strategy adapted from Tian 2020.

**Taxanes and Germ Cell - PubMed**	
Male	((male[tiab] OR males OR boy OR boys OR boyfriend OR boyhood OR man OR men))
Taxanes	AND ((((((((((((paclitaxel) OR docetaxel) OR taxus) OR taxol) OR taxotere) OR taxane) OR cabazitaxel) OR Jevtana) OR kabazitaxel) OR 33069-62-4) OR 114977-28-5) OR 183133-96-2)
Childhood cancer	AND (((leukemia) OR leukaemia) OR leukemic*) OR leukaemic*) OR leukaemi*) OR childhood ALL) OR AML) OR lymphoma) OR lymphom*) OR Hodgkin) OR hodgkin*) OR T-cell) OR B-cell) OR non-hodgkin) OR sarcoma) OR sarcom*) OR sarcoma) OR Ewing's) OR Ewing*) OR osteosarcoma) OR osteosarcom*) OR wilms tumor) OR wilms*) OR nephroblastom*) OR neuroblastoma) OR neuroblastom*) OR rhabdomyosarcoma) OR rhabdomyosarcoma*) OR teratoma) OR teratom*) OR hepatoma) OR hepatom*) OR hepatoblastoma) OR hepatoblastom*) OR PNET) OR Medulloblastoma) OR medulloblastom*) OR PNET*) OR Neuroectodermal tumors, primitive) OR Retinoblastoma) OR retinoblastom*) OR meningioma) OR meningiom*) OR glioma) OR gliom*) OR pediatric oncology) OR paediatric oncology) OR childhood cancer) OR childhood tumor) OR childhood tumors) OR brain tumor*) OR brain tumour*) OR brain neoplasms) OR central nervous system neoplasm) OR central nervous system neoplasms) OR central nervous system tumor*) OR central nervous system tumour*) OR brain cancer*) OR brain neoplasm*) OR intracranial neoplasm*) OR testis neoplasm) OR neoplasm, testicular) OR testicular neoplasm) OR testicular neoplasms) OR testis cancer) OR testicular cancer) OR testis tumor) OR testicular cancer) OR cancer of testis) OR testis neoplasm*) OR testis tumour*) OR testis tumor*) OR leukemia, lymphocytic, acute)
Germ cell	AND ((Spermatogenesis) OR gonadal disorder) OR spermiogenesis) OR spermatocytogenesis) OR spermatogenic failure) OR azoospermia) OR oligospermia) OR asthenozoospermia) OR teratozoospermia) OR oligoasthenoteratozoospermia) OR dysspermia) OR normozoospermic) OR semen) OR semen analysis) OR semen quality) OR sperm) OR sperm count) OR sperm motility) OR spermatozoa) OR progeny) OR offspring) OR posterity) OR fertility) OR infertility) OR subfertility) OR reproduction) OR fertilization) OR conception) OR paternity) OR fatherhood) OR parenthood) OR pregnancy outcome) OR fertile) OR infertile) OR subfertile) OR sperm maturation) OR aspermia) OR spermatozoon abnormality) OR germ cell) OR spermatogonia) OR spermatogonial) OR spermatogonium) OR meiosis) OR gonocyte) OR spermatid) OR spermatids) OR follicle stimulating hormone) OR FSH)

**Taxanes and Germ Cell – Embase**	
Male	((male.ti,ab,kw. OR males OR boy OR boys OR boyfriend OR boyhood OR man OR men))
Ifosfamide/ cyclophosphamide	AND ((((((((((((paclitaxel) OR docetaxel) OR taxus) OR taxol) OR taxotere) OR taxane) OR cabazitaxel) OR Jevtana) OR kabazitaxel) OR 33069-62-4) OR 114977-28-5) OR 183133-96-2)
Childhood cancer	AND (((leukemia) OR leukaemia) OR leukemic*) OR leukaemic*) OR leukaemi*) OR childhood ALL) OR AML) OR lymphoma) OR lymphom*) OR Hodgkin) OR hodgkin*) OR T-cell) OR B-cell) OR non-hodgkin) OR sarcoma) OR sarcom*) OR sarcoma) OR Ewing's) OR Ewing*) OR osteosarcoma) OR osteosarcom*) OR wilms tumor) OR wilms*) OR nephroblastom*) OR neuroblastoma) OR neuroblastom*) OR rhabdomyosarcoma) OR rhabdomyosarcoma*) OR teratoma) OR teratom*) OR hepatoma) OR hepatom*) OR hepatoblastoma) OR hepatoblastom*) OR PNET) OR Medulloblastoma) OR medulloblastom*) OR PNET*) OR Neuroectodermal tumors, primitive) OR Retinoblastoma) OR retinoblastom*) OR meningioma) OR meningiom*) OR glioma) OR gliom*) OR pediatric oncology) OR paediatric oncology) OR childhood cancer) OR childhood tumor) OR childhood tumors) OR brain tumor*) OR brain tumour*) OR brain neoplasms) OR central nervous system neoplasm) OR central nervous system neoplasms) OR central nervous system tumor*) OR central nervous system tumour*) OR brain cancer*) OR brain neoplasm*) OR intracranial neoplasm*) OR testis neoplasm) OR neoplasm, testicular) OR testicular neoplasm) OR testicular neoplasms) OR testis cancer) OR testicular cancer) OR testis tumor) OR testicular cancer) OR cancer of testis) OR testis neoplasm*) OR

	testis tumour*) OR testis tumor*) OR leukemia, lymphocytic, acute)
Germ cell	AND ((Spermatogenesis) OR gonadal disorder) OR spermiogenesis) OR spermatocytogenesis) OR spermatogenic failure) OR azoospermia) OR oligospermia) OR asthenozoospermia) OR teratozoospermia) OR oligoasthenoteratozoospermia) OR dysspermia) OR normozoospermic) OR semen) OR semen analysis) OR semen quality) OR sperm) OR sperm count) OR sperm motility) OR spermatozoa) OR progeny) OR offspring) OR posterity) OR fertility) OR infertility) OR subfertility) OR reproduction) OR fertilization) OR conception) OR paternity) OR fatherhood) OR parenthood) OR pregnancy outcome) OR fertile) OR infertile) OR subfertile) OR sperm maturation) OR aspermia) OR spermatozoon abnormality) OR germ cell) OR spermatogonia) OR spermatogonial) OR spermatogonium) OR meiosis) OR gonocyte) OR spermatid) OR spermatids) OR follicle stimulating hormone) OR FSH)

**Taxanes and Sertoli Cell - PubMed**	
Male	((male[tiab] OR males OR boy OR boys OR boyfriend OR boyhood OR man OR men))
Taxanes	AND ((((((((((((paclitaxel) OR docetaxel) OR taxus) OR taxol) OR taxotere) OR taxane) OR cabazitaxel) OR Jevtana) OR kabazitaxel) OR 33069-62-4) OR 114977-28-5) OR 183133-96-2)
Childhood cancer	AND (((leukemia) OR leukaemia) OR leukemic*) OR leukaemic*) OR leukaemi*) OR childhood ALL) OR AML) OR lymphoma) OR lymphom*) OR Hodgkin) OR hodgkin*) OR T-cell) OR B-cell) OR non-hodgkin) OR sarcoma) OR sarcom*) OR sarcoma) OR Ewing's) OR Ewing*) OR osteosarcoma) OR osteosarcom*) OR wilms tumor) OR wilms*) OR nephroblastom*) OR neuroblastoma) OR neuroblastom*) OR rhabdomyosarcoma) OR rhabdomyosarcoma*) OR teratoma) OR teratom*) OR hepatoma) OR hepatom*) OR hepatoblastoma) OR hepatoblastom*) OR PNET) OR Medulloblastoma) OR medulloblastom*) OR PNET*) OR Neuroectodermal tumors, primitive) OR Retinoblastoma) OR retinoblastom*) OR meningioma) OR meningiom*) OR glioma) OR gliom*) OR pediatric oncology) OR paediatric oncology) OR childhood cancer) OR childhood tumor) OR childhood tumors) OR brain tumor*) OR brain tumour*) OR brain neoplasms) OR central nervous system neoplasm) OR central nervous system neoplasms) OR central nervous system tumor*) OR central nervous system tumour*) OR brain cancer*) OR brain neoplasm*) OR intracranial neoplasm*) OR testis neoplasm) OR neoplasm, testicular) OR testicular neoplasm) OR testicular neoplasms) OR testis cancer) OR testicular cancer) OR testis tumor) OR testicular cancer) OR cancer of testis) OR testis neoplasm*) OR testis tumour*) OR testis tumor*) OR leukemia, lymphocytic, acute)
Sertoli cell	AND ((((((Sertoli) OR anti-mullerian) OR AMH) OR inhibin) OR inhibin B) OR androgen receptor))

**Taxanes and Sertoli Cell - Embase**	
Male	((male.ti,ab,kw. OR males OR boy OR boys OR boyfriend OR boyhood OR man OR men))
Taxanes	AND ((((((((((((paclitaxel) OR docetaxel) OR taxus) OR taxol) OR taxotere) OR taxane) OR cabazitaxel) OR Jevtana) OR kabazitaxel) OR 33069-62-4) OR 114977-28-5) OR 183133-96-2)
Childhood cancer	AND (((leukemia) OR leukaemia) OR leukemic*) OR leukaemic*) OR leukaemi*) OR childhood ALL) OR AML) OR lymphoma) OR lymphom*) OR Hodgkin) OR hodgkin*) OR T-cell) OR B-cell) OR non-hodgkin) OR sarcoma) OR sarcom*) OR sarcoma) OR Ewing's) OR Ewing*) OR osteosarcoma) OR osteosarcom*) OR wilms tumor) OR wilms*) OR nephroblastom*) OR neuroblastoma) OR neuroblastom*) OR rhabdomyosarcoma) OR rhabdomyosarcoma*) OR teratoma) OR teratom*) OR hepatoma) OR hepatom*) OR hepatoblastoma) OR hepatoblastom*) OR PNET) OR Medulloblastoma) OR medulloblastom*) OR PNET*) OR Neuroectodermal tumors, primitive) OR Retinoblastoma) OR retinoblastom*) OR meningioma) OR meningiom*) OR glioma) OR gliom*) OR pediatric oncology) OR paediatric oncology) OR childhood cancer) OR childhood tumor) OR childhood tumors) OR brain tumor*) OR brain tumour*) OR brain neoplasms) OR central nervous system neoplasm) OR central nervous system neoplasms) OR central nervous system tumor*) OR central nervous system tumour*) OR brain cancer*) OR brain neoplasm*) OR intracranial neoplasm*) OR testis neoplasm) OR neoplasm, testicular) OR testicular neoplasm) OR testicular neoplasms) OR testis cancer) OR testicular cancer) OR testis tumor) OR testicular cancer) OR cancer of testis) OR testis

	neoplasm*) OR testis tumour*) OR testis tumor*) OR leukemia, lymphocytic, acute)
Sertoli cell	AND (((((((Sertoli) OR anti-mullerian) OR AMH) OR inhibin) OR inhibin B) OR androgen receptor))

Taxanes and Leydig Cell - PubMed	
Male	((male[tiab] OR males OR boy OR boys OR boyfriend OR boyhood OR man OR men))
Taxanes	AND ((((((((((((paclitaxel) OR docetaxel) OR taxus) OR taxol) OR taxotere) OR taxane) OR cabazitaxel) OR Jevtana) OR kabazitaxel) OR 33069-62-4) OR 114977-28-5) OR 183133-96-2)
Childhood cancer	AND (((leukemia) OR leukaemia) OR leukemic*) OR leukaemic*) OR leukaemi*) OR childhood ALL) OR AML) OR lymphoma) OR lymphom*) OR Hodgkin) OR hodgkin*) OR T-cell) OR B-cell) OR non-hodgkin) OR sarcoma) OR sarcom*) OR sarcoma) OR Ewing's) OR Ewing*) OR osteosarcoma) OR osteosarcom*) OR wilms tumor) OR wilms*) OR nephroblastom*) OR neuroblastoma) OR neuroblastom*) OR rhabdomyosarcoma) OR rhabdomyosarcoma*) OR teratoma) OR teratom*) OR hepatoma) OR hepatom*) OR hepatoblastoma) OR hepatoblastom*) OR PNET) OR Medulloblastoma) OR medulloblastom*) OR PNET*) OR Neuroectodermal tumors, primitive) OR Retinoblastoma) OR retinoblastom*) OR meningioma) OR meningiom*) OR glioma) OR gliom*) OR pediatric oncology) OR paediatric oncology) OR childhood cancer) OR childhood tumor) OR childhood tumors) OR brain tumor*) OR brain tumour*) OR brain neoplasms) OR central nervous system neoplasm) OR central nervous system neoplasms) OR central nervous system tumor*) OR central nervous system tumour*) OR brain cancer*) OR brain neoplasm*) OR intracranial neoplasm*) OR testis neoplasm) OR neoplasm, testicular) OR testicular neoplasm) OR testicular neoplasms) OR testis cancer) OR testicular cancer) OR testis tumor) OR testicular cancer) OR cancer of testis) OR testis neoplasm*) OR testis tumour*) OR testis tumor*) OR leukemia, lymphocytic, acute)
Leydig cell	AND ((((((((((((((((((((((((((((androgen hormone insufficiency) OR leydig cell) OR leydig failure) OR testicular failure) OR interstitial cell failure) OR gonadal failure) OR hypogonadism) OR low testosterone) OR testosterone deficiency) OR leydig cell insufficiency) OR androgen deficiency) OR low testosterone*) OR hypogonadism*) OR leydig cell*) OR testosterone) OR luteinising hormone) OR LH) OR steroidogenesis) OR puberty) OR pubertal) OR testicular volume) OR testis volume) OR tanner stage) OR tanner staging) OR androgen) OR androgens) OR androgenic))

Taxanes and Leydig Cell - Embase	
Male	((male.ti,ab,kw. OR males OR boy OR boys OR boyfriend OR boyhood OR man OR men))
Taxanes	AND ((((((((((((paclitaxel) OR docetaxel) OR taxus) OR taxol) OR taxotere) OR taxane) OR cabazitaxel) OR Jevtana) OR kabazitaxel) OR 33069-62-4) OR 114977-28-5) OR 183133-96-2)
Childhood cancer	AND (((leukemia) OR leukaemia) OR leukemic*) OR leukaemic*) OR leukaemi*) OR childhood ALL) OR AML) OR lymphoma) OR lymphom*) OR Hodgkin) OR hodgkin*) OR T-cell) OR B-cell) OR non-hodgkin) OR sarcoma) OR sarcom*) OR sarcoma) OR Ewing's) OR Ewing*) OR osteosarcoma) OR osteosarcom*) OR wilms tumor) OR wilms*) OR nephroblastom*) OR neuroblastoma) OR neuroblastom*) OR rhabdomyosarcoma) OR rhabdomyosarcoma*) OR teratoma) OR teratom*) OR hepatoma) OR hepatom*) OR hepatoblastoma) OR hepatoblastom*) OR PNET) OR Medulloblastoma) OR medulloblastom*) OR PNET*) OR Neuroectodermal tumors, primitive) OR Retinoblastoma) OR retinoblastom*) OR meningioma) OR meningiom*) OR glioma) OR gliom*) OR pediatric oncology) OR paediatric oncology) OR childhood cancer) OR childhood tumor) OR childhood tumors) OR brain tumor*) OR brain tumour*) OR brain neoplasms) OR central nervous system neoplasm) OR central nervous system neoplasms) OR central nervous system tumor*) OR central nervous system tumour*) OR brain cancer*) OR brain neoplasm*) OR intracranial neoplasm*) OR testis neoplasm) OR neoplasm, testicular) OR testicular neoplasm) OR testicular neoplasms) OR testis cancer) OR testicular cancer) OR testis tumor) OR testicular cancer) OR cancer of testis) OR testis neoplasm*) OR testis tumour*) OR testis tumor*) OR leukemia, lymphocytic, acute)
Leydig cell	AND ((((((((((((((((((((((((((((androgen hormone insufficiency) OR leydig cell) OR leydig failure) OR testicular failure) OR interstitial cell failure) OR gonadal failure) OR hypogonadism) OR low testosterone) OR testosterone deficiency) OR leydig cell

	insufficiency) OR androgen deficiency) OR low testosterone*) OR hypogonadism*) OR leydig cell*) OR testosterone) OR luteinising hormone) OR LH) OR steroidogenesis) OR puberty) OR pubertal) OR testicular volume) OR testis volume) OR tanner stage) OR tanner staging) OR androgen) OR androgens) OR androgenic))
