# Supplementary material for: Impact of taxane-based chemotherapeutics on male reproductive function
Source: Reprod Fertil. 2023 Mar 28;4(1):e220134. doi: 10.1530/RAF-22-0134 (PMC10083651; doi:10.1530/RAF-22-0134)
Supplement: Supplementary Table 2: Full text articles screened and excluded [file supplementary_table_2.pdf]

Supplementary Table 2: Full text articles screened and excluded

Study	Title	Journal	Volume	Issue	Pages	DOI / PubMed ID
Badreldin 2016	The efficacy of irinotecan, paclitaxel, and oxaliplatin (IPO) in relapsed germ cell tumours with high-dose chemotherapy as consolidation: a non-cisplatin-based induction approach	BJU International	117	3	418-23	DOI: 10.1111/bju.13004
Batra 2019	Early experience with chemotherapy intensification for poor prognosis metastatic germ cell cancer and unfavorable tumor marker decline	Canadian Urological Association Journal	14	2	43-47	DOI: 10.5489/cuaj.5802
Beyer 1996	Phase II study of paclitaxel in patients with relapsed or cisplatin-refractory testicular cancer	Annals of Oncology	7	1	31-34	DOI: 10.1093/oxfordjournals.annonc.a010473
Bokemeyer 1994	Preliminary results of a phase I/II trial of paclitaxel in patients with relapsed or cisplatin-refractory testicular cancer	Journal of Cancer Research & Clinical Oncology	120	12	754-7	DOI: 10.1007/BF01194278
Bokemeyer 2008	Combination chemotherapy with gemcitabine, oxaliplatin, and paclitaxel in patients with cisplatin-refractory or multiply relapsed germ-cell tumors: a study of the German Testicular Cancer Study Group	Ann Oncol	19	3	448-53	DOI: 10.1093/annonc/mdm526
Chiappori 2008	Phase I/II study of atrasentan, an endothelin A receptor antagonist, in combination with paclitaxel and carboplatin as first-line therapy in advanced non-small cell lung cancer	Clin Cancer Res	14	5	1464-9	DOI: 10.1158/1078-0432.CCR-07-1508
Chovanec 2014	Adenocarcinoma of the rete testis - a rare case of testicular malignancy	Clin Oncol	27	2	136-7	DOI: 10.14735/amko2014136
DeBacker 2006	Testicular germ cell tumors in children: Management and outcome in a series of 20 patients	J Pediatr Urol	2	3	197-201	DOI: 10.1016/j.jpurol.2005.08.001
DeGiorgi 2004	Weekly gemcitabine, paclitaxel, oxaliplatin combination chemotherapy in patients with Cisplatin-refractory germ cell tumor: preliminary experience	Am J Clin Oncol	27	5	457-60	DOI: 10.1097/01.coc.0000128727.40450.9e
DePasquale 2020	Salvage treatment for children with relapsed/refractory germ cell tumors: The Associazione Italiana Ematologia Oncologia Pediatrica (AIEOP)	Pediatric Blood and Cancer	67	3	e28125	DOI: 10.1002/pbc.28125

	experience					
deWit 1999	Management of intermediate-prognosis germ-cell cancer: results of a phase I/II study of Taxol-BEP	International Journal of Cancer	83	6	831-3	DOI: 10.1002/(sici)1097-0215(19991210)83:6<831::aid-ijc24>3.0.co;2-o
Eggener 2007	Pathologic findings and clinical outcome of patients undergoing retroperitonial lymph node dissection after multiple chemotherapy regimens for metastatic testicular germ cell tumors	Cancer	109	3	528-535	DOI: 10.1002/cncr.22440
Einhorn 2007	Phase II study of paclitaxel plus gemcitabine salvage chemotherapy for germ cell tumors after progression following high-dose chemotherapy with tandem transplant	J Clin Oncol	25	5	513-6	DOI: 10.1200/JCO.2006.07.7271
Feldman 2013	Phase II trial of paclitaxel, ifosfamide, and cisplatin (TIP) for previously untreated patients (pts) with intermediate-or poor-risk germ cell tumors (GCT)	Journal of Clinical Oncology	31	6 SUPPL. 1		DOI: 10.1200/jco.2013.31.6_suppl.336
Feldman 2015	Phase I/II Trial of Paclitaxel With Ifosfamide Followed by High-Dose Paclitaxel, Ifosfamide, and Carboplatin (TI-TIC) With Autologous Stem Cell Reinfusion for Salvage Treatment of Germ Cell Tumors	Clin Genitourin Cancer	13	5	453-60	DOI: 10.1016/j.clgc.2015.05.003
Finlay 2015	Preliminary results of a feasibility pilot study of "gempox" (gemcitabine, oxaliplatin, and paclitaxel) in pediatric and adult patients with refractory or recurrent central nervous system (CNS) germcell tumors (GCT): The international CNS GCT consortium trial, CNS GCT-4	Neuro-Oncology	17	SUPPL. 3		DOI: 10.1093/neuonc/nov061.37
Fizazi 2014	Personalised chemotherapy based on tumour marker decline in poor prognosis germ-cell tumours (GETUG 13): a phase 3, multicentre, randomised trial	Lancet Oncol	15	13	1442-1450	DOI: 10.1016/S1470-2045(14)70490-5

Gamulin 2020	Testicular cancer- between de-escalation and high-dose chemotherapy with peripheral blood stem-cell transplantation	Libri Oncologici	48	SUPPL 1	56-57	Web link: https://www.hok2020.com/wp-content/uploads/2020/09/13.HOK_.LO_compressed.pdf
Gerl 1996	Antitumor activity of paclitaxel after failure of high-dose chemotherapy in a patient with late relapse of a non-seminomatous germ cell tumor	Anti-Cancer Drugs	7	6	716-8	DOI: 10.1097/00001813-199608000-00014
Hamid 2019	Autologous Stem-Cell Transplantation Outcomes for Relapsed Metastatic Germ-Cell Tumors in the Modern Era	Clin Genitourin Cancer	17	1	58-64.e1	DOI: 10.1016/j.clgc.2018.09.009
Hara 2005	High dose chemotherapy including paclitaxel (T-ICE) combined with peripheral blood stem cell transplantation for male germ cell tumor. Preliminary report	International Journal of Urology	12	12	1074-8	DOI: 10.1111/j.1442-2042.2005.01235.x
Hartmann 2007	Phase I/II study of sequential dose-intensified ifosfamide, cisplatin, and etoposide plus paclitaxel as induction chemotherapy for poor prognosis germ cell tumors by the German Testicular Cancer Study Group	J Clin Oncol	25	36	5742-7	DOI: 10.1200/JCO.2007.11.9099
Hinton 2002	Phase II study of paclitaxel plus gemcitabine in refractory germ cell tumors (E9897): a trial of the Eastern Cooperative Oncology Group	J Clin Oncol	20	7	1859-63	DOI: 10.1200/JCO.2002.07.158
Ishioka 2007	Incorporation of TIP (paclitaxel, ifosfamide, cisplatin) into first-line therapy for intermediate to poor risk testicular germ cell tumors with unfavorable marker decline after initial two cycles chemotherapy: a report of three cases	International Journal of Urology	14	5	455-7	DOI: 10.1111/j.1442-2042.2007.01755.x
Ji 2021	Clinical experience of male primary choriocarcinoma at the samsung medical center	Cancer Research and Treatment	53	3	874-880	DOI: 10.4143/crt.2020.1066
Kawai 2003	Paclitaxel, ifosfamide and cisplatin regimen is feasible for Japanese patients with advanced germ cell cancer	Jpn J Clin Oncol	33	3	127-31	DOI: 10.1093/jjco/hyg029

Kojima 2015	Identification of a subgroup with worse prognosis among patients with poor-risk testicular germ cell tumor	International Journal of Urology	22	10	923-7	DOI: 10.1111/iju.12844
Kondagunta 2005	Combination of paclitaxel, ifosfamide, and cisplatin is an effective second-line therapy for patients with relapsed testicular germ cell tumors	J Clin Oncol	23	27	6549-55	DOI: 10.1200/JCO.2005.19.638
Kumano 2007	First-line high-dose chemotherapy combined with peripheral blood stem cell transplantation for patients with advanced extragonadal germ cell tumors	International Journal of Urology	14	4	336-338	DOI: 10.1111/j.1442-2042.2006.01718.x
Lewin 2014	High-dose chemotherapy with autologous stem cell transplantation in relapsed or refractory germ cell tumours: outcomes and prognostic variables in a case series of 17 patients	Intern Med J	44	8	771-8	DOI: 10.1111/imj.12486
Lian 2019	Clinical Benefit of Sorafenib Combined with Paclitaxel and Carboplatin to a Patient with Metastatic Chemotherapy-Refractory Testicular Tumors	Oncologist	24	12	e1437-e1442	DOI: 10.1634/theoncologist.2019-0295
LigiaCebotaru 2016	A phase II single institution single arm prospective study with paclitaxel, ifosfamide and cisplatin (TIP) as first-line chemotherapy in high-risk germ cell tumor patients with more than ten years follow-up and retrospective correlation with ERCC1, Topoisomerase 1, 2A, p53 and HER-2 expression	J buon	21	3	698-708	PMID: 27569093
Lin 2021	Cervical malignant teratoma masquerading as a hematoma: a case report	Journal of International Medical Research	49	2		DOI: 10.1177/0300060520984597
Liu 2013	Preliminary results of a prospective feasibility pilot study of "GEMPOX" (gemcitabine, oxaliplatin, and paclitaxel) in patients with refractory or recurrent CNS germ cell tumours	British Journal of Neurosurgery	27	4	e22-e23	· DOI: 10.3109/02688697.2013.801247
Lotz 2005	Sequential high-dose chemotherapy protocol for relapsed poor prognosis germ cell tumors combining two mobilization and cytoreductive treatments followed by three high-dose chemotherapy regimens supported by autologous stem cell transplantation. Results of the phase II multicentric TAXIF trial	Ann Oncol	16	3	411-8	DOI: 10.1093/annonc/mdi087

Mardiak 2005	Gemcitabine plus cisplatin and paclitaxel (GCP) in second-line treatment of germ cell tumors (GCT): a phase II study	Neoplasma	52	3	243-7	PMID: 15875087
Mardiak 2005	Paclitaxel plus ifosfamide and cisplatin in second-line treatment of germ cell tumors: a phase II study	Neoplasma	52	6	497-501	PMID: 1628469
Mardiak 2007	Paclitaxel, bleomycin, etoposide, and cisplatin (T-BEP) as initial treatment in patients with poor-prognosis germ cell tumors (GCT): a phase II study	Neoplasma	54	3	240-5	PMID: 17447857
Marwaha 2007	The importance of the dose of etoposide in the initial treatment of metastatic germ cell tumors and advances in management of patients that relapse	Canadian Journal of Urology	14	5	3692-6	PMID: 17949524
Mascia 2015	Testicular cancer: Clinical features in a retrospective survey analysis of a single institution of sardinia	Anticancer Research	35	6	3717-3718	DOI: 10.1093/annonc/mdv341.44
McNeish 2004	Paclitaxel-containing high-dose chemotherapy for relapsed or refractory testicular germ cell tumours	Br J Cancer	90	6	1169-75	DOI: 10.1038/sj.bjc.6601664
Mead 2005	A phase II trial of TIP (paclitaxel, ifosfamide and cisplatin) given as second-line (post-BEP) salvage chemotherapy for patients with metastatic germ cell cancer: a medical research council trial	Br J Cancer	93	2	178-84	DOI: 10.1038/sj.bjc.6602682
Motzer 1994	Phase II trial of paclitaxel shows antitumor activity in patients with previously treated germ cell tumors	J Clin Oncol	12	11	2277-83	DOI: 10.1200/JCO.1994.12.11.2277
Motzer 1997	Paclitaxel in salvage therapy for germ cell tumors	Semin Oncol	24	5 Suppl 15	S15-83-s15-85	PMID: 9346229
Motzer 2000	Paclitaxel (Taxol) combination therapy for resistant germ cell tumors	Semin Oncol	27	1 Suppl 1	33-5	PMID: 1069704
Motzer 2000	Paclitaxel, ifosfamide, and cisplatin second-line therapy for patients with relapsed testicular germ cell cancer	J Clin Oncol	18	12	2413-8	DOI: 10.1200/JCO.2000.18.12.2413
Mulherin 2015	Long-term survival with paclitaxel and gemcitabine for germ cell tumors after progression following high-dose chemotherapy with tandem transplant	Am J Clin Oncol	38	4	373-6	DOI: 10.1097/COC.0b013e31829e19e0

Nakamura 2015	Importance of continuous sequential chemotherapy and multimodal treatment for advanced testicular cancer: a high-volume Japanese center experience	Medicine (Baltimore)	94	11	e653	DOI: 10.1097/MD.0000000000000653
Narayan 2016	Risk-Stratified Initial Salvage Therapy for Relapsed or Refractory Metastatic Germ Cell Tumors	Clin Genitourin Cancer	14	6	524-529	DOI: 10.1016/j.clgc.2016.03.015
Necchi 2014	Combination of paclitaxel, cisplatin, and gemcitabine (TPG) for multiple relapses or platinum-resistant germ cell tumors: long-term outcomes	Clin Genitourin Cancer	12	1	63-69.e1	DOI: 10.1016/j.clgc.2013.07.005
Nicolai 2009	Long-term results of a combination of paclitaxel, cisplatin and gemcitabine for salvage therapy in male germ-cell tumours	BJU International	104	3	340-6	DOI: 10.1111/j.1464-410X.2009.08453.x
Nieto 2005	Phase I and pharmacokinetic study of docetaxel combined with melphalan and carboplatin, with autologous hematopoietic progenitor cell support, in patients with advanced refractory malignancies	Biol Blood Marrow Transplant	11	4	297-306	DOI: 10.1016/j.bbmt.2005.01.002
Nitta 2016	A Case of Mixed Germ Cell Tumor in the Intramedullary Spinal-cord	Tokai J Exp Clin Med	41	3	147-51	PMID: 27628608
Nonomura 2007	Paclitaxel, ifosfamide, and nedaplatin (TIN) salvage chemotherapy for patients with advanced germ cell tumors	International Journal of Urology	14	6	527-31	DOI: 10.1111/j.1442-2042.2006.01702.x
Oechsle 2011	Long-term survival after treatment with gemcitabine and oxaliplatin with and without paclitaxel plus secondary surgery in patients with cisplatin-refractory and/or multiply relapsed germ cell tumors	Eur Urol	60	4	850-5	DOI: 10.1016/j.eururo.2011.06.019
Pamenter 2003	Bilateral testicular cancer: A preventable problem? Experience from a large cancer centre	BJU International	92	1	43-46	DOI: 10.1046/j.1464-410x.2003.04285.x
Park 2011	Salvage chemotherapy with paclitaxel, ifosfamide, and cisplatin (TIP) in relapsed or cisplatin-refractory germ cell tumors	Onkologie	34	08-Sep	416-20	DOI: 10.1159/000331129
Pashankar 2018	Treatment of refractory germ cell tumors in children with paclitaxel, ifosfamide, and carboplatin: A report from the Children's	Pediatric Blood and Cancer	65	8	e27111	DOI:10.1002/pbc.27111

	Oncology Group AGCT0521 study					
Passos-Coelho 2011	Suboptimal survival of male germ-cell tumors in southern Portugal-a population-based retrospective study for cases diagnosed in 1999 and 2000	Annals of Oncology	22	5	1215-1220	DOI: 10.1093/annonc/mdq551
Pectasides 2010	Methotrexate, paclitaxel, ifosfamide, and cisplatin in poor-risk nonseminomatous germ cell tumors	Urol Oncol	28	6	617-23	DOI: 10.1016/j.urolonc.2008.10.013
Pont 1997	Chemotherapy for germ cell tumors relapsing after high-dose chemotherapy and stem cell support: a retrospective multicenter study of the Austrian Study Group on Urologic Oncology	Ann Oncol	8	12	1229-34	DOI: 10.1023/a:1008286305312
Rick 2001	Salvage treatment with paclitaxel, ifosfamide, and cisplatin plus high-dose carboplatin, etoposide, and thiotepa followed by autologous stem-cell rescue in patients with relapsed or refractory germ cell cancer	J Clin Oncol	19	1	81-8	DOI: 10.1200/JCO.2001.19.1.81
Ronnen 2005	Incidence of late-relapse germ cell tumor and outcome to salvage chemotherapy	J Clin Oncol	23	28	6999-7004	DOI: 10.1200/JCO.2005.21.956
Rosenthal 2009	Phase III multi-institutional trial of adjuvant chemotherapy with paclitaxel, estramustine, and oral etoposide combined with long-term androgen suppression therapy and radiotherapy versus long-term androgen suppression plus radiotherapy alone for high-risk prostate cancer: preliminary toxicity analysis of RTOG 99-02	International Journal of Radiation Oncology, Biology, Physics	73	3	672-8	DOI: 10.1016/j.ijrobp.2008.05.020
Sadeghi 2013	Phase II study of gemcitabine, oxaliplatin, and paclitaxel (GOT) on a 2-weekly schedule in patients (pts) with refractory germ cell tumor (rGCT): Final results	Journal of Clinical Oncology	31	15 SUPPL. 1		DOI: 10.1200/jco.2013.31.15_suppl.4531

Sandler 1998	A phase II trial of paclitaxel in refractory germ cell tumors	Cancer	82	7	1381-6	DOI: 10.1002/(sici)1097-0142(19980401)82:7<1381::aid-cncr23>3.0.co;2-1
Seidel 2016	Efficacy and safety of gemcitabine, oxaliplatin, and paclitaxel in cisplatin-refractory germ cell cancer in routine care--Registry data from an outcomes research project of the German Testicular Cancer Study Group	Urol Oncol	34	4	167.e21-8	DOI: 10.1016/j.urolonc.2015.11.007
Seymour 1999	Secondary acute myeloid leukemia with inv(16): report of two cases following paclitaxel-containing chemotherapy and review of the role of intensified ara-C therapy	Leukemia	13	11	1735-40	DOI: 10.1038/sj.leu.2401552.
Shamash 2007	A phase II study using a topoisomerase I-based approach in patients with multiply relapsed germ-cell tumours	Ann Oncol	18	5	925-30	DOI: 10.1093/annonc/mdm002
Shatara 2020	Final report of the prospective NEXT/CNS-GCT-4 consortium trial (gempox followed by marrowablative chemotherapy) in patients with refractory/ recurrent CNS germ cell tumors	Neuro-Oncology	22	SUPPL 3		DOI: 10.1093/neuonc/noaa222.283
Shiraishi 2009	Salvage chemotherapy with paclitaxel and gemcitabine plus nedaplatin (TGN) as part of multidisciplinary therapy in patients with heavily pretreated cisplatin-refractory germ cell tumors	Int J Clin Oncol	14	5	436-41	DOI: 10.1007/s10147-009-0899-y
Shiraishi 2018	Chemotherapy for metastatic testicular cancer: The first nationwide multi-institutional study by the Cancer Registration Committee of the Japanese Urological Association	International Journal of Urology	25	8	730-736	DOI: 10.1111/iju.13720
Sonnichsen 1994	Saturable pharmacokinetics and paclitaxel pharmacodynamics in children with solid tumors	J Clin Oncol	12	3	532-8	DOI: 10.1200/JCO.2001.19.1.81

Sottotetti 2015	A case of metastatic adenocarcinoma of submandibular gland with hyperexpression of androgen and HER2 receptor: A "target driven" therapeutic strategy	Italian Journal of Medicine	9	SUPPL. 2	101-102	Web link: file:///Users/mrimmer2/Downloads/pgranata,+IJM_2015_(s2)_bassa.pdf
Stemmer 1996	High-dose paclitaxel, cyclophosphamide, and cisplatin with autologous hematopoietic progenitor-cell support: a phase I trial	J Clin Oncol	14	5	1463-72	DOI: 10.1200/JCO.1996.14.5.1463
Tanaka 2010	Long-term outcome of chemotherapy for advanced testicular and extragonadal germ cell tumors: A single-center 27-year experience	Japanese Journal of Clinical Oncology	40	1	73-78	DOI: 10.1093/jjco/hyp121
Theodore 2008	A phase II multicenter study of oxaliplatin in combination with paclitaxel in poor prognosis patients who failed cisplatin-based chemotherapy for germ-cell tumors	Ann Oncol	19	8	1465-1469	DOI: 10.1093/annonc/mdn122
Tran 2017	Treatment and outcomes of central nervous system nongerminomatous germ cell tumors with early relapse during induction chemotherapy	Pediatric Neurology	71		106	DOI: 10.1093/neuonc/noy059.258
Tryakin 2011	Paclitaxel+BEP (T-BEP) regimen as induction chemotherapy in poor prognosis patients with nonseminomatous germ cell tumors: a phase II study	Urology	78	3	620-5	DOI: 10.1016/j.urology.2011.05.005
Vozianov 2014	High dose chemotherapy in patients with germ cell testicular tumors with unfavorable prognosis	European Urology, Supplements	13	6	e1243	DOI: 10.1093/annonc/mdu485
Weiss 2017	A phase Ib study of pembrolizumab plus chemotherapy in patients with advanced cancer (PembroPlus)	Br J Cancer	117	1	33-40	DOI: 10.1038/bjc.2017.145
Yamada 2013	Salvage chemotherapy with docetaxel, ifosfamide and nedaplatin (DIN) for patients with advanced germ cell tumors: a preliminary report	Jpn J Clin Oncol	43	7	734-9	DOI: 10.1093/jjco/hyt060
